# Chronic Obstructive Pulmonary Disease, inflammation and co-morbidity – a common inflammatory phenotype?

**DOI:** 10.1186/1465-9921-7-70

**Published:** 2006-05-02

**Authors:** Martin J Sevenoaks, Robert A Stockley

**Affiliations:** 1 Department of Medicine, Queen Elizabeth Hospital Birmingham, UK

## Abstract

Chronic Obstructive Pulmonary Disease (COPD) is and will remain a major cause of morbidity and mortality worldwide. The severity of airflow obstruction is known to relate to overall health status and mortality. However, even allowing for common aetiological factors, a link has been identified between COPD and other systemic diseases such as cardiovascular disease, diabetes and osteoporosis.

COPD is known to be an inflammatory condition and neutrophil elastase has long been considered a significant mediator of the disease. Pro-inflammatory cytokines, in particular TNF-α (Tumour Necrosis Factor alpha), may be the driving force behind the disease process. However, the roles of inflammation and these pro-inflammatory cytokines may extend beyond the lungs and play a part in the systemic effects of the disease and associated co-morbidities. This article describes the mechanisms involved and proposes a common inflammatory TNF-α phenotype that may, in part, account for the associations.

## Introduction

Chronic Obstructive Pulmonary Disease (COPD) is and will remain a major cause of morbidity and mortality Worldwide [1]. The severity of the airflow obstruction as assessed by the forced expired volume in 1 second (FEV_1_) is a predictor of overall health status [2] and mortality from both respiratory disease [3] and all causes [4].

Recently interest has arisen because of the association of COPD with other systemic diseases including cardiovascular disease [5], diabetes [6], osteoporosis [7] and peptic ulceration [8]. Whereas these associations may represent common aetiological factors such as cigarette smoking and steroid usage, careful studies allowing for these factors have still identified an unexplained link.

COPD is an inflammatory condition and by-products of the inflammatory process lead to the tissue damage and physiological adaptations that typify the condition. The association with smoking is well known although only a proportion of smokers (typically attributed to about 15%) develop clinically important airflow obstruction suggesting a genetic predisposition. In this respect elastase released from activated neutrophils has long been considered to be a significant mediator of the disease [9]. Recent extensive studies involving the smoking mouse model have confirmed this to be a major mechanism possibly driven by pro-inflammatory cytokines of which tumour necrosis factor-alpha (TNF-α) appears to be central [10].

However, the roles of inflammation and these pro-inflammatory cytokines have been proposed to extend beyond the lung in COPD. In particular, they are thought to play a key role in the muscle wasting related to severe emphysema and possibly other co-morbidities. This article describes the mechanisms involved and proposes a common TNF-α driven physiological process that may, in part, account for the associations.

### COPD and systemic inflammation

Initially, it was thought that the establishment of lung inflammation resulted in an "overspill" into the circulation producing a low-grade systemic inflammation. However, soluble tumour necrosis factor receptor (sTNF-R) or Interleukin-8 (IL-8) in sputum and plasma do not correlate [11] suggesting that a simple overspill explanation is not correct.

Patients with COPD have higher baseline levels of several circulating inflammatory markers [12]. The reasons are not clear and it remains unknown whether the systemic inflammation is a primary or secondary phenomenon. Specific subsets of patients with COPD have been identified and those with increased resting energy expenditure and decreased fat-fee mass have more marked elevation of stable state C reactive protein (CRP) and lipopolysaccharide binding protein [13]. Furthermore, those with higher levels of systemic inflammation lack a response to nutritional supplementation [14], raising the possibility that this may be an associated phenomenon rather than cause and effect.

Both COPD and smoking have been shown to have negative effects on markers of oxidative stress. Smoking and acute exacerbations of COPD resulted in a marked imbalance in redox status [15]. Raised levels of lipid peroxidation products confirm the persistence of increased oxidative stress and other markers have also been elevated [16]. The increase in oxidative stress may result in the inactivation of antiproteases, airspace epithelial damage, mucus hypersecretion, increased influx of neutrophils into lung tissue and the expression of pro-inflammatory mediators [17,18].

Changes have also been noted in various inflammatory cells in peripheral blood, including neutrophils and lymphocytes [19]. Patients with COPD have increased numbers of neutrophils in the lungs, increased activation of neutrophils in peripheral blood and an increase in TNF-α and sTNF-R. It has been suggested that this indicates the importance of a TNF-α/neutrophil axis in maintaining the COPD phenotype [20,21].

The central role of TNF-α in lung inflammation is not only supported by animal models [10] but has also been implicated in the COPD phenotype with low body mass index [7]. Cytokine production by macrophages is enhanced by hypoxia in vitro [22] and thus the inverse correlation between arterial oxygen tension and circulating TNF-α and sTNF-R may be the result of systemic hypoxia [22]. It is tempting therefore to assume that TNF inhibition would be as beneficial in COPD as it has been in other inflammatory conditions such as rheumatoid arthritis and Crohn's disease [23,24]. However, this was also hypothesised for congestive heart failure (CHF). TNF-α is believed to play a key role in the pathogenesis of CHF and raised levels are associated with a higher mortality in CHF [25]. However, studies using TNF-α blockade have shown no benefit and possibly an increase in mortality for reasons that are not clear [26], suggesting it is not just a simple cause and effect.

### Muscle wasting

Low body mass index (BMI), age, and low arterial oxygen tension have been shown to be significant independent predictors of mortality in COPD [27,28]. More specifically, loss of fat-free mass (FFM) adversely affects respiratory and peripheral muscle function, exercise capacity and health status. Both weight loss and loss of FFM appear to be the result of a negative energy balance, and are seen more commonly in emphysema [29].

In starvation and nutritional imbalance there is an adaptive reduction in resting energy requirements [30]. In contrast (as in cachexia) increased resting energy expenditure has been noted in many COPD patients, linked to systemic inflammation [13,31]. Furthermore nutritional intake is also generally adequate (apart from during acute exacerbations). The traditional view that this increased basal metabolic rate is due to an increased oxygen consumption by respiratory muscles has been shown to be only part of the reason [32]. Whilst there is no universally agreed definition of cachexia (derived from the Greek *kakos *[bad] and *hexis *[condition]), accelerated loss of skeletal muscle in the context of a chronic inflammatory response is a characteristic feature [33], and not limited to COPD. Patients with cachexia display preferential loss of FFM, enhanced protein degradation [34] and poor responsiveness to nutritional interventions [35,36]. In addition, cachectic patients exhibit changes in the metabolism of proteins, lipids and carbohydrates that are thought to be related to systemic rather than local inflammation [36,37]. Thus muscle wasting in COPD displays similarities to the cachexia seen in chronic heart failure, renal failure, acquired immunodeficiency syndrome and cancer (amongst others). The importance of cachexia in these conditions is not only that it is associated with reduced survival [35,38-40], but also that it is related to poor functional status and health-related quality of life [33]. Common findings in all these conditions include increased levels of circulating pro-inflammatory molecules including TNF-α, IL-1, IL-6, IL-8, interferon-γ (INF-γ) and reduced levels of anabolic hormones including insulin-like growth factors and testosterone [33].

**Figure 1:**
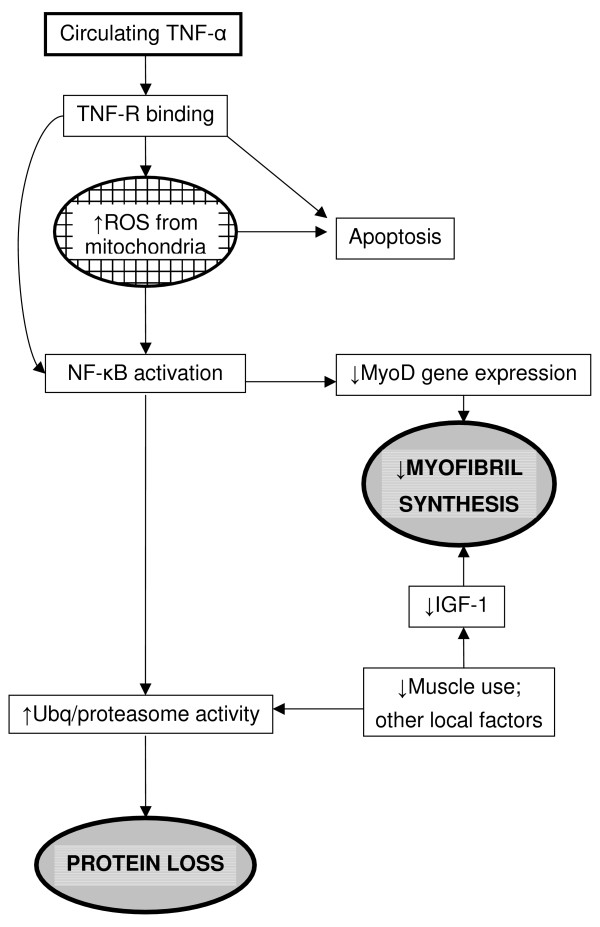
Pathogenic process implicated in muscle wasting in COPD. Circulating TNF-α present in some patients with COPD binds to peripheral muscle cell receptors stimulating the production of ROS and apoptosis. In addition the receptor binding stimulates NF-κB activation, possibly enhanced by ROS. Protein loss is caused directly via increased ubiquitin activity, and indirectly via decreased MyoD expression decreasing myofibril synthesis. Protein loss is amplified by a reduction in muscle use. This is the result of a reduction in IGF-1 production (leading to a decrease in myofibril synthesis), and an increase in ubiquitin activity. TNF-α – tumour necrosis factor alpha TNFR – tumour necrosis factor receptor ROS – reactive oxygen species NF-κB – nuclear factor kappa beta Ubq – ubiquitin IGF – insulin-like growth factor

TNF-α plays a central role in the muscle wasting and weight loss seen in COPD. It has several direct effects (anorexia, altered levels of circulating hormones and catabolic cytokines, and altered end organ sensitivities to them) which could promote muscle wasting [41] predominantly via the ubiquitin pathway. This process is mediated by nuclear factor-κB (NF-κB), a transcription factor that is inactive when bound to its inhibitor but which can be activated by inflammatory cytokines including TNF-α [42]. In muscle cells NF-κB can interfere with skeletal muscle differentiation and repair via inhibition of MyoD expression [43](Figure 1).

Oudijk et al [20] proposed three different mechanisms by which TNF-α could induce muscle loss. Firstly, protein loss can be directly stimulated in the skeletal muscle cells. Secondly, apoptosis can be stimulated through various signalling pathways via interaction with the TNF-α receptors on the muscle cells. Thirdly, reactive oxygen species (ROS) can lead to changes in TNF-α/NF-κB signalling, although the implications of such changes in this pathway have yet to be clarified. Nevertheless, it appears that inflammation and ROS have a synergistic action on muscle breakdown [37] and since COPD is associated with increased oxidant stress [44] it is likely that this factor also plays a role.

### Diabetes

A common process may explain why patients with COPD have a 1.8 RR of developing type II diabetes [45]. Epidemiological studies have provided evidence that indicators of inflammation can predict the development of diabetes and glucose disorders [6,46]. Indeed, in the ARIC study fibrinogen, circulating white blood cells count and lower serum albumin predicted the development of type II diabetes [6]. Furthermore patients with non insulin dependant diabetes mellitus have increased circulating levels of TNF-α, IL-6 and CRP [47]. For these reasons the roles of circulating cytokines in the pathogenesis of diabetes and insulin resistance have received increasing interest. Adipose tissue secretes numerous adipokines which markedly influence lipid and glucose/insulin metabolism. These include TNF-α and an antagonist, the "protective", adipose tissue specific, adiponectin.

Sonnenberg and colleagues [48] proposed that TNF-α might be a mediator of the diabetic process. As described above, this cytokine acts via its receptor to activate the nuclear transcription factor NF-κB leading to cytokine production, up regulation of adhesion molecules and increasing oxidative stress. Indeed, this latter effect together with TNF-α may provide a stimulating pathway that interferes with glucose metabolism and insulin sensitivity. This pathway can be antagonised by adiponectin which reduces NF-κB activation [49].

This concept is supported by several clinical and experimental observations. Firstly, it is known that TNF-α expression is increased in patients with weight gain and insulin resistance [50]. Perhaps this represents a modulating effect as TNF-α stimulates lipolysis [51] but TNF-α levels are associated with hyper insulinaemia and insulin resistance [52]. Other studies have also confirmed that an acute phase response (CRP) is increased in obesity and associated with insulin resistance [53]. Furthermore, adiponectin levels are reduced in obesity and associated with insulin resistance and hyper insulinaemia [54]. However, the most direct supporting data for this putative axis comes from the obese, insulin resistant mouse where TNF-α inhibition improves insulin sensitivity [50].

**Figure 2:**
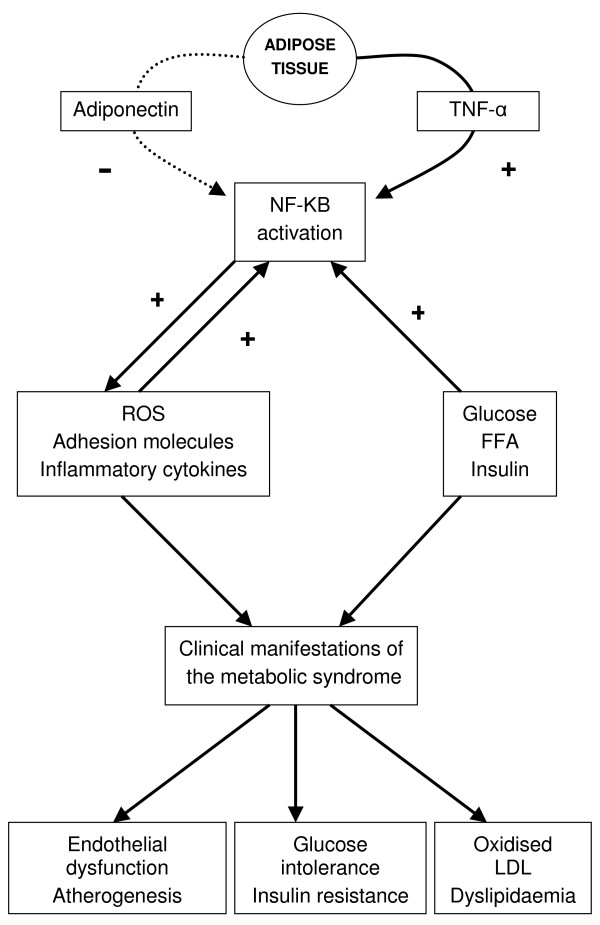
The roles of TNF-α, adiponectin and NF-κB in the metabolic syndrome. [Adapted from Sonnenberg et al (41)] TNF-α secreted from adipose tissue in conjunction with circulating glucose, FFA and insulin stimulate NF-κB activation. This action is opposed by adiponectin (indicated by the broken line), also secreted from adipose tissue. Activation of the PPARγ pathway (for example by TZDs) has been shown to directly increase expression of adiponectin and reduce TNF-α. Further activation of NF-κB is induced through the resulting increase in inflammatory cytokines, adhesion molecules and oxidative stress, leading to the clinical manifestations of the metabolic syndrome. The metabolic syndrome is a constellation of cardiovascular risk factors that is associated with a trebling of risk of type 2 diabetes and a doubling of risk of cardiovascular disease. Several definitions have been proposed [80-83] leading to some confusion and differences in prevalence rates. The International Diabetes Federation have recently proposed a practical, globally applicable definition of the syndrome using waist circumference plus any two of raised triglycerides, reduced HDL-cholesterol, raised blood pressure and raised fasting plasma glucose [84]. TNF-α – tumour necrosis factor alpha NF-κB – nuclear factor kappa beta FFA – free fatty acid LDL – low-density lipoprotein PPARγ – peroxisome proliferator activated receptor gamma TZD – thiazolidenedione

These observations support the concept that inflammation as reflected in acute phase proteins are in some way intimately associated with the development of glucose intolerance and insulin resistance. This concept is summarized in figure 2 which is derived from the proposal of Sonnenberg et al [48].

Whereas these studies still raise the issue of cause and effect there have been attempts at proof of concept. Thiazolidinediones are agonists for peroxisome proliferator-activated receptor gamma (PPARγ) – a ligand-activated transcription factor belonging to the nuclear hormone receptor superfamily. This class of drug not only decreases inflammatory markers including TNF-α, soluble ICAM-1, fibrinogen, MIP1 and CRP but also improves insulin action [55-58]. These studies are thus in keeping with a common inflammatory process/pathway linking COPD and type II diabetes. They are also consistent with the predictive role of acute phase proteins in the development of type II diabetes [6].

Fernandez-Real [59] expanded on this process to relate the inflammatory mechanism of insulin resistance to atherosclerosis where similar hypotheses have been proposed.

### Atherosclerosis

Ridker et al [60] recently published data indicating that baseline CRP showed a concentration dependant relative risk for future cardiovascular events. Pai et al [61] assessed the risk of coronary heart disease and related this to the circulating levels of several inflammatory markers. The authors found that high levels of CRP and IL-6 were significantly related to an increased risk in both males and females. The relative risk was 1.79 for individuals whose baseline was greater than 3 mg/L.

**Figure 3:**
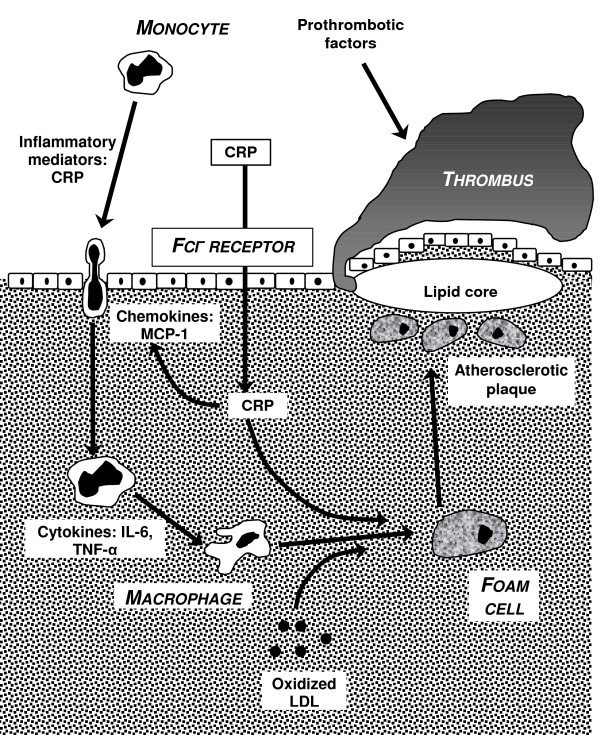
The inflammatory processes involved in atherosclerotic plaque formation. CRP binds to endothelial cells via the Fcγ receptor and is internalized, facilitating monocyte binding via the production of MCP-1. Further activation leads to further cytokine release and differentiation of the monocytes into macrophages. In the presence of oxidized LDL, CRP aids the production of foam cells – the basis of an atherosclerotic plaque. CRP – C reactive protein TNF-α – tumour necrosis factor alpha IL-6 – interleukin-6 MCP1 – monocyte chemotactic protein 1 LDL – low density lipoprotein ROS – reactive oxygen species

C-reactive protein is a type I acute phase protein with properties suggesting it is an archaic form of immunity which possesses the ability to bind to bacteria subsequently facilitating the binding of complement necessary for bacterial killing and/or phagocytosis. The protein can increase up to 1000 fold within days of the commencement of an inflammatory process. TNF-α, IL-1 and IL-6 stimulate CRP synthesis by inducing hepatic gene expression [62], implicating TNF-α at the core of the process. CRP is known to bind and cause lattice formation and precipitation leading to passive haemaglutination. Macrophages have receptors for CRP and CRP can increase cytokine production [63,64]. These features may be central to atheroma production. C-reactive protein may deposit directly on to the arterial wall during atherogenesis, possibly via the Fcgamma (Fcγ) receptor [65] facilitating monocyte adherence through the production of the monocyte chemokine MCP-1. Further activation can result in production of other pro-inflammatory cytokines and differentiation of the monocytes into macrophages (Figure 3).

In the presence of oxidised low density lipoproteins, CRP can facilitate the production of foam cells which are the building blocks of atherosclerotic plaques (figure 3).

Recent studies by Smeeth et al [66] have indicated that the risk of having a myocardial infarct or cerebrovascular event are increased greatly within the first 3 days after an "acute systemic respiratory tract infection", defined by the authors as pneumonia, acute bronchitis, "chest infections" or influenza (4.95 RR for myocardial infarct and 3.19 RR for stroke). These events are accompanied by a well recognised acute inflammatory response and cytokine production. Indeed in patients with COPD not only is the baseline CRP over 3 mg/L in almost half of the patients but the further rise during an acute exacerbation [67] is also associated with a rise in fibrinogen [68] increasing the pro thrombotic risk. This may well account for the increased risk of vascular events in COPD and particularly the likelihood of the increased mortality within a few month of hospital admission for an acute exacerbation [69].

### Osteoporosis

The risk of osteoporosis with steroid use is well known, but patients with COPD have an increased risk even in the absence of steroid use. McEvoy and colleagues [70] observed that vertebral fractures were present in up to 50% steroid naive males with COPD. More recently studies by Bolton et al confirmed that osteopoenia was a feature of COPD and associated with an increase in circulating TNF-α [7]. Again, the association suggests a cause and effect.

Post menopausal osteoporosis is related to high serum levels of TNF-α and IL-6 [71]. It is known that macrophages can differentiate into osteoclasts in the presence of marrow mesenchymal cells. These latter cells release the cytokine RANK ligand (RANKL) which is a member of the TNF-α superfamily. TNF-α and IL-1 enhance this process and can induce RANKL expression in marrow stromal cells and synergise with RANKL in osteoclastogenesis [72], although osteoclast formation can also be induced by IL-6, independent of RANKL [73]. However, other inflammatory conditions such as rheumatoid arthritis [74] and periodontal disease [75] have T cells induced to produce RANKL and it is therefore likely that a similar process occurs in COPD.

The role of pro-inflammatory cytokines may therefore be central to the osteoporosis associated with inflammatory disease. In support of this concept is the study reported by Gianni et al [71] who confirmed that Raloxifene was able to decrease TNF-α transcription and serum levels whilst increasing bone density. Again these data support a close association between the pro-inflammatory processes and osteopoenia.

### Peptic ulceration

Finally peptic ulceration is known to be more frequent in patients with chronic bronchitis and emphysema [76]. Furthermore, studies in patients with gastric ulcers have found a decrease in FEV_1 _and vital capacity in smokers and non-smokers [8]. More recently Roussos and colleagues [77] demonstrated that helicobacter sero-positivity is increased in COPD patients to 77.8% (compared to 54% in control subjects). Furthermore they noted that sero-positivity to the greater pro-inflammatory phenotype expressing CaGA was present in 53.9% of patients compared to 29.3% of controls. Once more, although these associations could represent common factors such as smoking and socio-economic status, the authors hypothesised that the chronic activation of inflammatory mediators induced by H pylori could amplify the development of COPD. The increased prevalence of the CaGA positive strain supports this hypothesis as it can stimulate the release of IL-1 and TNF-α [78] that may enhance the endothelial adhesion and migration of inflammatory cells into the lung. Whether such a process enhances the inflammatory response to cigarette smoke in the lungs remains unknown. An alternative suggested by the authors is that overspill inhalation of H pylori or its exotoxins into the lungs may in their own right lead to chronic airway inflammation and hence tissue damage. There is, however, no direct evidence of this in COPD, although the hypothesis is feasible and testable by using eradication therapy and observing the subsequent decline in lung function in COPD.

## Conclusion

**Figure 4:**
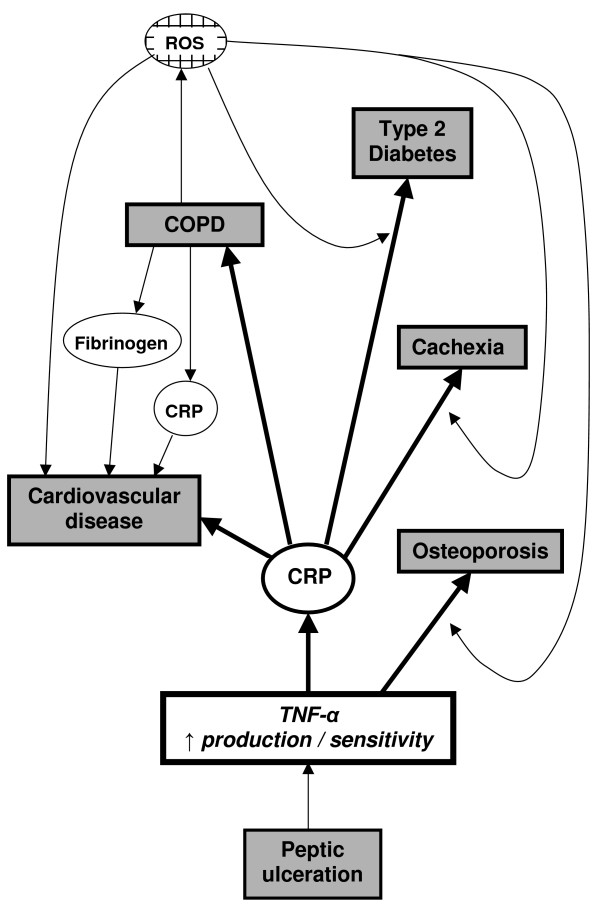
The central role of TNF-α in co-morbidity associated with COPD. TNF-α appears to play a central role in the pathogenesis of COPD and other conditions that are increasingly being recognised as systemic inflammatory diseases. Certain TNF-α receptor polymorphisms are associated with increased severity of disease [85,86] and this may be due to enhanced TNF-α effects. CRP levels can be increased directly by TNF-α and other cytokines. Elevated CRP levels appear to be particularly crucial in the pathogenesis of cardiovascular disease. ROS released as a result of COPD may enhance the likelihood of developing cardiovascular disease, diabetes and osteoporosis. TNF-α – tumour necrosis factor – alpha CRP – C reactive protein ROS – reactive oxygen species

In summary several disease entities occur more commonly in the presence of each other and are associated with similar inflammatory pathophysiology suggesting that a common process results in the clinical overlap. TNF-α appears to be a central mediator in this process suggesting that factors influencing its production may lead to a cascade of events, making several conditions more likely (Figure 4). COPD may enhance this phenomenon by the associated release of ROS. Alternatively it is possible that the systemic inflammatory response to COPD precipitates disease processes at distant sites in its own right, although this seems less likely. Whatever the relationship, it does suggest that COPD patients may present to other specialties because of the co-morbidity. Furthermore, the diagnosis may be missed because of common symptomatology (dyspnoea as a result of cardiovascular disease or obesity). As effective anti-inflammatory therapy becomes available for COPD it will be of importance not only to monitor the effect on the lungs but also any associated co-morbidities. This may explain why inhaled corticosteroids in COPD are associated with decreased cardiovascular mortality [79] but clearly further studies are warranted to dissect this process in detail.

## Abbreviations

All abbreviations are expanded in the text

## Competing interests

The author(s) declare that they have no competing interests.

## Authors' contributions

MJS and RAS co-authored the paper
